# Evidence of secular trends during the COVID-19 pandemic in a stepped wedge cluster randomized trial examining sexual and reproductive health outcomes among Indigenous youth

**DOI:** 10.1186/s13063-023-07223-1

**Published:** 2023-04-01

**Authors:** Michael Anastario, Elizabeth Rink, Paula Firemoon, Nicole Carnegie, Olivia Johnson, Malory Peterson, Ana Maria Rodriguez

**Affiliations:** 1 Robert Stempel College of Public Health & Social Work, Florida International University, ACH5 11200 SW 8th St, Office 415, Miami, FL 33174 USA; 2 Montana State University, Bozeman, MT USA; 3 Fort Peck Community College, Poplar, MT USA; 4 The Public Health Company, Bozeman, MT USA

**Keywords:** Stepped wedge design, Secular trend, American Indians, Sexual health, COVID-19 pandemic

## Abstract

**Background:**

Nen ŨnkUmbi/EdaHiYedo (“We Are Here Now,” or *NE*) is an intervention to prevent STIs, HIV, HCV, and teen pregnancy among Assiniboine and Sioux youth of the Fort Peck Reservation in the state of Montana in the USA. A cluster-randomized stepped-wedge design (SWD) trial is used to evaluate *NE*, where clusters are schools. The purpose of this study is to evaluate whether there is evidence of a secular trend associated with the COVID-19 pandemic.

**Methods:**

The original study design is a cluster-randomized stepped-wedge design (SWD), in which five schools that youth from Fort Peck attend are the clusters to be randomized into the intervention one at a time, with all schools eventually being randomized to the intervention across three steps. N/E is a 5-year study involving 456 15- to 18-year-old youth. For this study, we use a mixed quantitative and qualitative methods approach to understand how the COVID-19 pandemic may have been associated with the study’s primary outcome variables. Data were drawn from the first cluster exposed to the intervention and one control cluster that did not yet receive the intervention during the period in which COVID-19 mitigation efforts were being implemented. A pre-post COVID questionnaire was added to core measures administered, and semistructured qualitative interviews were conducted with youths regarding their perceptions of how the pandemic altered their sexual behaviors.

**Results:**

One hundred eighteen youth responded to the questionnaire and 31 youth participated in semistructured qualitative interviews. Youth reporting having sex with less people due to COVID-19 reported more sex acts (incident rate ratio (IRR)=3.6, 95% CI 1.6–8.1) in comparison to those who did not report having sex with less people, and youth who reported having sex with the same amount of people due to COVID-19 reported less sex acts (IRR=0.31, 95% CI 0.14–0.7) in comparison to those who did not report having sex with the same amount of people. Youth reporting having sex less times due to COVID-19 experienced a greater number of sex acts in comparison to those who did not report having sex less times (IRR=2.7, 1.2–6.4). Results suggest that more sexually active individuals reported perceiving having sex with less people and less frequent engagement in sex during the pandemic. It is possible that the COVID-19 pandemic period was associated with a truncation in the distribution of sexual activity that would bias an estimate of the intervention’s effect.

**Conclusion:**

Findings suggest evidence of a secular trend. This trend must be accounted for at trial end, and sensitivity analyses are recommended. Documenting and reporting on these findings encourages transparent reporting during the implementation of a SWD trial during a global pandemic, and informs endline analyses.

**Trial registration:**

This trial is registered with the Clinical trials registry of the US National Library of Medicine at the National Institutes of Health (NIH). It was registered on October 1, 2018. The study presented in this manuscript is funded by NIH National Institute on Minority Health and Health Disparities (NIMHD), Award # R01MD012761-01, Elizabeth Rink (Principal Investigator). The study’s ClinicalTrials.gov number is NCT03694418.

**Supplementary Information:**

The online version contains supplementary material available at 10.1186/s13063-023-07223-1.

## Background

Nen ŨnkUmbi/EdaHiYedo (“We Are Here Now,” or *NE*) was proposed as an intervention to prevent STIs, HIV, HCV, and teen pregnancy among Assiniboine and Sioux youth of the Fort Peck Reservation in the state of Montana in the USA. Funding for the trial was provided by the US National Institutes of Health Minority Health and Health Disparities in 2018, and the research was projected to occur over a 5-year period. A cluster-randomized stepped-wedge design (SWD) is being used to evaluate *NE*. The protocol for the original SWD trial is published elsewhere [1]. The unprecedented global COVID-19 pandemic and the mitigation strategies enacted in response to the pandemic, including school closures, social distancing measures, and stay-in-place orders, disrupted the planned implementation of the trial and likely had effects on the study’s primary outcomes [2]. The examination presented in this paper is concerned with understanding whether the COVID-19 pandemic may have been associated with secular trends in the study’s primary outcome. Findings will be informative for future analyses that attempt to estimate the intervention’s effect.

There is now a growing body of literature concerning the impact of COVID-19 on clinical trials [3–7]. Complex, trial-specific issues spanning recruitment, communication with staff and key stakeholders, intervention delivery, and data collection are unfolding in unprecedented ways [8]. For SWD trials, where clusters are sequentially randomized to the intervention in steps, community-level rollout during the current COVID-19 pandemic will confront factors that may confound estimates of intervention effectiveness including social distancing measures, novel treatments for illness, and patient care interventions [9]. In the context of *NE*, where schools are the clusters randomized to the intervention, COVID mitigation strategies such as school shutdowns and social distancing required adaptations, flexibility, and a focused attempt to understand them as they were unfolding. The original trial is registered with the Clinical trials registry of the US National Library of Medicine at the National Institutes of Health (NIH) (https://clinicaltrials.gov/ct2/show/NCT03694418). The study’s ClinicalTrials.gov number is NCT03694418. Trial modifications are not yet posted in the registration page. However, trial adaptations in response to the COVID-19 pandemic have been published elsewhere [2].

The COVID-19 pandemic was an unexpected, exogenous factor that could affect numerous aspects of an ongoing SWD trial, including secular trends in the primary outcome. SWD trials experience risks of bias attributable to secular trends that are not specified in the analytic stage. The staggered rollout period may occur during a time in which an exogenous factor or event significantly impacts the outcome and/or sample and may partially confound the estimated effect of the intervention. In addition, SWD trials with a small number of heterogeneous clusters may experience added risk for secular trends that are easy to miss during the analytic stage [10]. Furthermore, attrition associated with the pandemic has been considered a factor that could jeopardize trial completion [6]. Prospectively documenting the impact of an external event that clearly presents itself (such as the COVID-19 pandemic) is a rare opportunity for informing future analytic strategies regarding the intervention’s effectiveness.

This current study focuses on sexual and reproductive health outcomes that may have been affected by the COVID-19 pandemic during a SWD trial. To evaluate *NE*, a cluster randomized SWD was determined to be most appropriate given that it was a rigorous method for evaluating the efficacy of the intervention while simultaneously being a straightforward way to meet tribal members’ requests that all students participating in the study receive the potential benefits of the intervention. The trial was designed prior to the COVID-19 pandemic, which represented a threat to the trial by producing a potential secular trend associated with the primary outcome variable. The community’s contribution to decision-making regarding trial design occurred in the trial’s broader context of ongoing community-based participatory research (CBPR) [11].

### Objectives

The purpose of the examination presented in this paper is to understand whether the COVID-19 pandemic may have produced a secular trend affecting the trial’s primary outcome. Reporting these changes encourages transparent reporting and will be useful to others who are using cluster randomized controlled trial designs to evaluate similar outcomes.

## Methods

### Trial design

*N/E* is a cluster-randomized stepped-wedge design (SWD), in which five schools that youth from Fort Peck attend are the clusters to be randomized into the intervention one at a time, with all schools eventually being randomized to the intervention across three steps. N/E is a 5-year study involving 456 15- to 18-year-old youth. All clusters are observed at baseline, mid-trial, post-trial, and 3-month follow-up time points. N/E takes place on The Fort Peck Reservation, which is a rural space located in northeastern Montana, USA. Approximately 8000 enrolled tribal members, predominately from the Sioux and Assiniboine Nations, live on the 2.1-million-acre reservation space [12]. The five schools that serve as “clusters” are in separate communities within the reservation, mitigating the potential for cross-contamination.

N/E utilizes a CBPR strategy. CBPR has become an established methodological framework for partnering with Indigenous communities to conduct research, but has been sparsely applied to understanding and addressing Indigenous sexual and reproductive health (SRH) [13, 14]. CBPR also has the potential to facilitate the implementation of complex trials conducted in AI communities, particularly given its amenability to multi-sectoral stakeholder involvement in the research process [14–17]. The nexus of *NE's* trial design, grounded in a fusion of tribal requirements, local beliefs, and Western science, has an overarching framework of CBPR to address SRH disparities. *NE* is an example of an RCT that highlights the amassing research in prevention science with Indigenous communities [18–20]. This new era of RCT research emphasizes the integration of traditional knowledge systems, Indigenous culture, and western science to generate new standards of practice for RCTs with Indigenous communities [13, 21]. This highly generative process of integrating Indigenous perspectives with western research practices is aided by community-engaged methods such as CBPR. Because of *NE's* foundation in CBPR principles and practices, we were able to modify the study's trial design and meet tribal needs in response to the pandemic. We collected additional qualitative and quantitative data on potential secular trends to understand the ways in which the pandemic may have impacted the study’s outcome variables.

**Fig. 1:**
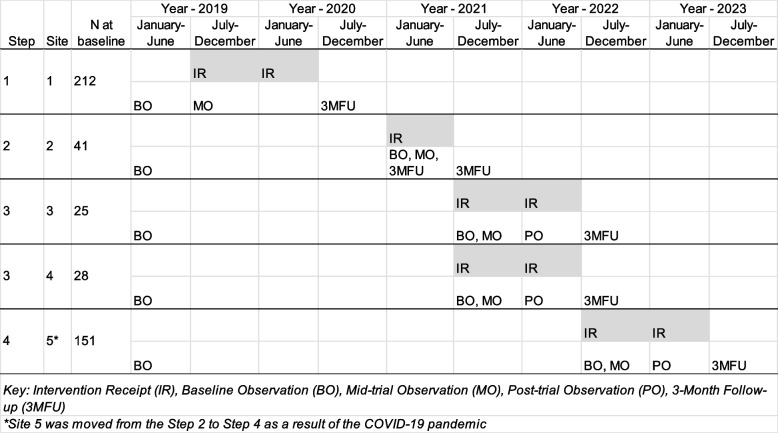
Stepped wedge design trial schematic

A more exhaustive description of COVID-19 trial modifications is provided elsewhere [2], a visual of the SWD trial schematic is provided in Fig. 1. In summary, the first cluster completed the intervention in February 2020, just before widespread COVID-19 pandemic mitigation strategies such as school closures and social distancing were implemented at Fort Peck. *NE's* implementation in the first cluster was nearing completion in Spring 2020 when the stay-at-home orders in Montana were implemented. At that point, *NE* had three remaining modules left of the full 18-module school-based curriculum and had Spring parent meetings planned. School closures began occurring in the middle of March 2020 at Fort Peck to mitigate coronavirus transmission. The school closures interrupted our ability to conduct a mid-trial assessment in the first cluster with student and parent participants. Due to the COVID-19 pandemic, a trial pause that lasted 90 days was initiated. A trial restart was possible if the second cluster that was originally randomized to the intervention be skipped. The choice to skip the cluster was made to mitigate the risk of exposure to COVID-19 for students in the larger second cluster, as well as protect the health of the Fort Peck research team who would have been required to travel to the other side of the reservation to implement the intervention. The subsequent cluster randomized to the intervention was physically closer to those delivering the intervention and collecting data, which mitigated risk by requiring less travel across the reservation. Based on baseline data, this shift would result in a projected 33% of the closed cohort sample changing order of intervention receipt (where a relatively large cluster was moved to the end of the intervention receipt sequence), and an addition of a fourth step to *NE* in order to accommodate conducting the intervention with this re-sequenced cluster.

### Participants

To be eligible for inclusion in N/E, students must be (1) 15 to 18 years old, (2) a registered member of a federally recognized tribe or an associate tribal member, and (3) a resident of Fort Peck with a parent/legal guardian. Exclusion criteria are minimal due to the community advisory board’s value of inclusion in N/E. Exclusion criteria include (1) not meeting the aforementioned inclusion criteria and (2) having a medically identified physical or cognitive impairment that would impede their understanding of and participation in the educational content and activities of Native Stand, Native Voices, and the cultural mentoring program. No persons who refuse to participate in the study, in whole or in part, will be coerced to engage in any study activity. After written consent and assent is obtained from the parent/legal guardian and child, respectively, they are enrolled in the study.

Student surveys for baseline, 3, and 9 months are administered during regularly scheduled classes by the tribal research director. Students receive $10 at the baseline, 3-month, and 9-month data collections and $20 at the 12-month data collection.

### Intervention

The original intervention design included a school-based SRH curriculum; a family-level curriculum, a cultural mentoring component at the community level, and a mobilizing strategy to activate a multi-sectoral network of youth-servicing organizations at the systems level in Fort Peck to coordinate SRH services for AI youth. The core delivery of *NE* was planned to last approximately 17 weeks. In the SWD trial, other clusters that had yet to receive the intervention and baseline observations for clusters prior to receipt of the intervention served as controls.

### Outcomes

The study’s primary outcome is sexual risk behavior, defined by a composite of the number of protected vaginal and sexual acts that students participated in during the 30 days preceding an interview, and the number of sexual acts during the 30 days preceding an interview. Outcomes data are collected through behavioral questionnaires administered to students using computer-assisted self-interviews.

During the pandemic, the study team did not conduct interviews via telephone or online. This was due to several of the study participants not having internet access in their homes. In some cases, data collection team members visited the homes of students who would complete the questionnaire while the data collector waited outside. In some cases, the students and parent/legal guardian would visit the research team’s office to complete the surveys.

### Sample size

We used a simulation-based power analysis to assess the study’s power. The estimated intra-class correlation from preliminary data is less than 0.001, but given the level of uncertainty in that estimate, we continued to use an intraclass correlation coefficient of 0.016 as found in a HIV-preventive intervention study for American Indian middle school youths [22]. Temporal autocorrelation between consecutive observations in the preliminary data is 0.45. We simulated a zero-inflated Poisson outcome via a two-stage approach, with a Bernoulli model to select participants who are not sexually active (with probability 0.6, as observed in preliminary data), and then simulating a partner count for those who are potentially active from a Poisson distribution with temporal and within-school correlation as above. We simulated the open-cohort design with accrual and attrition rates between time steps comparable to those observed in preliminary data. We varied the percent reduction in the outcome and assess the power to detect and effect of that size. Based on simulation results, the SWD trial has 80 percent power to detect a 34% in the number of sexual partners among youth.

### Randomization sequence generation

The first observation period occurred for all students across the five schools and served as the baseline measurement observation, in which none of the clusters had yet been randomized to the intervention condition. Following the single baseline observation, clusters were randomized to *N/E* and, following intervention receipt, were observed at mid-intervention, immediate post-intervention, and 3-month follow-up time periods. Confirmation of the final baseline survey collected was communicated to the team on May 30, 2019. The randomization procedure occurred on May 31, 2019. Schools were randomized to the intervention using a single sequence random assignment to clusters that determined the order of intervention receipt [23]. The study’s analyst randomized clusters to N/E following the first round of baseline data collection.

### Blinding

After assignment to interventions, the Principal Investigator, those responsible for delivering the intervention, and those responsible for collecting data are blinded from seeing the outcome variables over time.

### Analysis of secular trends

The purpose of this analysis is to understand whether the COVID-19 pandemic may have produced a secular trend affecting the trial’s primary outcome. Analyses of attrition and direct effects on the study’s outcomes are both informative to understanding the existence of a potential secular trend that could impact the trial.

#### Attrition

The rural and self-contained location of the intervention did not originally raise significant concerns regarding attrition, as the study team felt relatively confident in their ability to track students given normal school operations. However, the school closures and mitigation measures implemented in response to the pandemic resulted in appreciable attrition occurring within the first cluster receiving the intervention. Among the 212 students sampled at baseline in the first cluster that was randomized to the intervention, two students transferred to other schools that will receive the intervention after the baseline observation and were not considered in the present analysis, 60 students did not have an observation beyond baseline, and 71 students had a mid-intervention observation but no 3-month post-intervention observation (see Table 1). The first school randomized to the intervention had implemented a policy that students did not have to physically arrive to the school/site if parents did not want them to due to COVID-19. Several students were already being home schooled during the 3-month post-intervention observation. However, other unexpected quantitative patterns in attrition should nonetheless be explored and accounted for among the 210 students sampled in the first cluster at baseline.

**Table 1:** Attrition in the first school/cluster randomized to the intervention (*NE*)

Time period relative to cluster in the first step	Days since 1st baseline observation (range)	Total sample size for all individuals sampled at cluster		Sample size of closed cohort in cluster	
		Cluster exposed to intervention	Cluster unexposed to intervention	Cluster exposed to intervention	Cluster unexposed to intervention
Baseline	0–63	*n*=212	*n*=41	*n*=212	*n*=41
Mid-*NE*	265–267	*n*=187	--	*n*=145	--
3-months post *NE*	537–540	*n*=96	--	*n*=79	--
Second baseline	671	--	*n*=22	--	*n*=9

#### Effects associated with trial outcomes

In Spring 2020 at the onset of the COVID-19 pandemic, a series of COVID-related questions were added to the existing youth survey to measure the perceived impact of COVID-19 on SRH [2]. To respond to the possibility of there being an impact on secular trends, a *pre-post COVID questionnaire* was added to core measures administered to study participants. In close collaboration with the tribal community, the study team continued the trial while documenting deviations made in real time, which was a best practice recommended by leading research agencies worldwide to navigate the COVID-19 pandemic. All participants in the first cluster randomized to the intervention were given the questionnaire 3 months following intervention completion (*n*=96), and all participants from the second cluster (*n*=22) were given a second baseline questionnaire prior to receiving the intervention.

Questionnaire items were adapted from newly developed measures for COVID-19 [24]. Students were asked whether they quarantined at home with a parent, guardian, or other friends/relatives during the Montana stay-at-home order during the spring of 2020, and whether they accessed condoms, birth control, and tests during the spring of 2020. Students were also asked to self-report whether: they had sex with less/more/the same amount of people due to COVID; whether they had sex with less/more/the same frequency due to COVID; and whether they used condoms with less/more/the same frequency due to COVID.

In-depth qualitative interviews were conducted to better understand how the conditions of COVID-19 impacted everyday life at Fort Peck. Interviews were conducted with 31 youths across the reservation who had participated in the first baseline survey during the Spring of 2019. The scope of the qualitative interviews conducted was much larger than the subset of questions used to inform this current analysis. Interview questions and responses regarding the perceived impacts of COVID on sexual and other types of behaviors were extracted from transcripts to inform this current study. During the semi-structured interview, study participants were asked about their personal and friends' engagement in romantic and/or sexual relationships during the COVID-19 pandemic. Participants were asked: “Are you in a romantic and/or sexual relationship? If so, how the COVID-19 pandemic over the last year affected your relationship?” and “How did people in romantic relationships maintain their romantic relationships over the past year during COVID-19 pandemic?” among others. These questions were fielded in order to provide additional context regarding the study’s primary outcome variable.

### Statistical methods

#### Quantitative data analyses

For the analyses concerned with attrition, Random Forest (RF) algorithms were used to explore missing data patterns in the first cluster randomized to the intervention. RFs use recursive binary splitting to grow a tree on training data by segmenting the feature space into regions that minimize the classification error. RFs build de-correlated trees and average them, yielding a single consensus prediction [25]. The high variance produced by tree-based methods can be reduced with bagging (bootstrap aggregation). RFs can also accommodate a large number of predictor variables [26]. For missing data subsequent to each observation period in the first cluster (baseline, mid-intervention), 500 trees were developed with 16 variables ($\sqrt{p}$) tried at each node/split of a given tree. Variable importance measures (VIMs) were calculated for each predictor and used as a screening tool to rank and prioritize variables for subsequent follow-up. Logistic regression was used to aid in interpretation for variables with appreciable VIMs in both forests (baseline, mid-NE) that were developed.

For the analyses concerned with secular trends, the frequency of endorsement for COVID-related items was examined in relation to the number of sexual acts during the 30 days prior to the interview, and the number of protected acts of vaginal and anal sex in the 30 days prior to the interview. We conducted a test of overdispersion of the dependent variable by evaluating the *z* statistic, and chose to use negative binomial regression as opposed to Poisson regression [27]. In the negative binomial models, we offset the models for the number of sex acts by the number of sexual partners, and the models for the number of protected sex acts by the number of sex acts. We used the adjusted incident rate ratio (aIRR) to interpret the effects of the independent variables on the outcomes of interest. All analyses were conducted using STATA 14 [28].

#### Qualitative data analyses

Interview transcripts concerning romantic and sexual relationships were subject to an inductive analytic strategy [29]. First, line-by-line coding was conducted to generate a set of “open codes,” followed by a second round of “axial” coding to reduce the set of open codes to a manageable set of categories. A second coder applied axial codes to recode the open codes developed by the first coder. The overall inter-coder agreement was 94.3% and Cohen’s Kappa was 0.94. These values fall within a substantial range [30, 31].

## Results

### Attrition

RF algorithms for missing data at follow-up revealed high VIMs for *grade* and *age of student* in the baseline and midline observation periods for the first cluster randomized to the intervention (see Supplementary Material). Across the trees considered in the RF for the first cluster at baseline, grade and age of student showed the greatest variable importance compared to all other variables considered in the model. These patterns appeared in the RFs for both the baseline and mid-*NE* observation periods in the first cluster. Grade and age of student were used to develop propensity scores for missing data at follow-up in each observation period using a logistic regression model that included grade, age, and an interaction term between grade and age. Results from the models that illustrate the interaction effect are shown in the Supplementary Material.

### Secular trends — quantitative findings

There were no appreciable differences in endorsement of COVID-19-related items between the 96 participants who had completed the intervention, and the 22 participants who did not complete the intervention (see Table 2). There were associations between the self-assessed impacts of COVID-19 on sexual behaviors and self-reported sexual behaviors (Table 3). Individuals who reported accessing services from Indian Health Services for SRH items during the Spring of 2020 reported an increased number of sex acts (IRR=2.8, 95% CI 1.4–5.5) in comparison to those who did not access services. Individuals reporting having sex with less people due to COVID-19 reported more sex acts (IRR=3.6, 95% CI 1.6–8.1) in comparison to those who did not report having sex with less people, and individuals who reported having sex with the same amount of people due to COVID-19 reported less sex acts (IRR=0.31, 95% CI 0.14–0.7) in comparison to those who did not report having sex with the same amount of people. Individuals reporting having sex less times due to COVID-19 experienced a greater number of sex acts in comparison to those who did not report having sex less times (IRR=2.7, 1.2–6.4), and individuals reporting having sex the same amount of times reported fewer sex acts in comparison to those who did not report having sex the same amount of times (IRR=0.36, 95% CI 0.17–0.75). Individuals who reported having sex the same number of times due to COVID-19 experienced a 76% reduction in the number of protected acts of vaginal and/or anal sex (IRR=0.25, 95% CI 0.1–0.7). Individuals reporting that they used condoms more times due to COVID-19 reported a greater number of protected acts of vaginal and/or anal sex (IRR=4.6, 95% CI 1.2–17.6) in comparison to those who reported not using condoms more times due to COVID.

**Table 2:** Perceived effects of the COVID-19 pandemic in relation to intervention versus control group

Perceived effects of the COVID pandemic	COVID item endorsement by cluster	
	Intervention (*n*=96)	Control (*n*=22)
Quarantined at home during the Montana sheltering-in-place order during Spring 2020	82.1%	90.9%
Accessed services from IHS or tribal health services for SRH items^a^ during Spring 2020	21.3%	18.2%
Wanted or needed to access SRH items but could not	7.4%	13.6%
Concerned about contracting COVID-19 prevented access to SRH services	17.2%	13.6%
Worried about contracting COVID-19	64.2%	54.5%
Stayed at home more due to concern about contracting COVID-19	49.5%	72.7%
Had sex with less people due to COVID-19	6.4%	13.6%
Had sex with more people due to COVID-19	2.1%	0.0%
Had sex with the same amount of people due to COVID-19	91.5%	86.4%
Had sex less times due to COVID	9.6%	9.1%
Had sex more times due to COVID	4.3%	4.5%
Had sex the same amount of times due to COVID	86.2%	86.4%
Used condoms less times due to COVID	7.4%	4.8%
Used condoms more times due to COVID	6.4%	4.8%
Used condoms the same amount of times due to COVID	86.2%	90.5%

*Abbreviations:* *IHS* Indian Health Services, *SRH* sexual and reproductive health

^a^SRH items included condoms, birth control, a pregnancy test, or a STI test

**Table 3:** Perceived effects of the COVID-19 pandemic in relation to sexual behaviors

Perceived effects of the COVID pandemic	Average number of sex acts, (SD)		IRR^a^	(95% CI)	Average number of protected sex acts, (SD)		IRR^a^	(95% CI)
	Did not endorse item	Endorsed item			Did not endorse item	Endorsed item		
Quarantined at home during the Montana sheltering-in-place order during Spring 2020	1.2 (1.2)	.47 (1.2)	0.51	(.23–1.1)	.79 (1.4)	.22 (.82)	.36	(.11–1.1)
Accessed services from IHS or tribal health services for SRH items^b^ during Spring 2020	.37 (1.0)	1.5 (1.9)	2.8	(1.4–5.5)	.12 (.57)	1.0 (1.5)	2.7	(.85–8.4)
Wanted or needed to access SRH items but could not	.54 (1.3)	1.0 (1.6)	1.3	(.45–4.0)	.35 (.98)	0.0 (0.0)	0.0	(0.0–0.0)
Concerned about contracting COVID-19 prevented access to SRH services	.53 (1.3)	.84 (1.5)	1.0	(.41–2.5)	.29 (.91)	.42 (1.1)	1.0	(.25–4.1)
Worried about contracting COVID-19	.64 (1.5)	.55 (1.2)	.76	(.38–1.5)	.34 (1.0)	.30 (.89)	2.1	(.67–6.5)
Stayed at home more due to concern about contracting COVID-19	.48 (1.2)	.67 (1.4)	1.3	(.64–2.5)	.19 (.68)	.43 (1.1)	1.4	(.42–4.5)
Had sex with less people due to COVID-19	.41 (1.1)	2.4 (2.5)	3.6	(1.6–8.1)	.17 (.64)	1.9 (1.9)	1.4	(.40–5.2)
Had sex with more people due to COVID-19	.58 (1.3)	.5 (.71)	.82	(.06–12.0)	.31 (.95)	.50 (.71)	5.6	(.45–69.8)
Had sex with the same amount of people due to COVID-19	2.1 (2.4)	.41 (1.1)	.31	(.14–.70)	1.7 (1.8)	.16 (.64)	.53	(.15–1.9)
Had sex less times due to COVID	.43 (1.2)	1.9 (1.9)	2.7	(1.2–6.4)	.19 (.72)	1.4 (1.7)	2.4	(.74–7.8)
Had sex more times due to COVID	.54 (1.3)	1.4 (1.5)	2.0	.50–7.8)	.29 (.92)	1.0 (1.2)	3.1	(.66–14.7)
Had sex the same amount of times due to COVID	1.8 (1.8)	.38 (1.1)	.36	(.17–.75)	1.3 (1.6)	.15 (.67)	.24	(0.1–0.7)
Used condoms less times due to COVID	.45 (1.2)	1.6 (1.4)	2.5	(.83–7.4)	.26 (.84)	.63 (1.4)	.76	(.17–3.3)
Used condoms more times due to COVID	.51 (1.2)	.9 (1.2)	1.4	(.37–5.2)	.25 (.85)	.86 (1.2)	4.6	(1.22–17.6)
Used condoms the same amount of times due to COVID	1.3 (1.3)	.43 (1.2)	.47	(.19–1.1)	.73 (1.3)	.22 (.80)	.44	(.14–1.4)

*Abbreviations:* *IHS* Indian Health Services, *SRH* sexual and reproductive health, *IRR* incidence rate ratio, *CI* confidence interval, *SD* standard deviation

^a^IRRs were derived from a negative binomial regression model with the number of protected acts of vaginal and anal sex (past 30 days) as the dependent variable, with the model offset by the number of sexual acts (past 30 days)

^b^SRH items included condoms, birth control, a pregnancy test, or a STI test

### Secular trends — qualitative findings

A range of relationship experiences were described during the COVID-19 pandemic, ranging from no intimate interpersonal involvement to ongoing engagement in sex during the shelter-in-place period. Among those who were romantically involved or had friends who were romantically involved during the shelter-in-place period, sexual experiences varied widely. Axial codes are summarized in Table 4.

**Table 4:** Axial code descriptions derived from the qualitative analysis

**Axial code**	**Description of axial code**
CARING	Describing acts of self-care/caring for others (or not) during the pandemic across different areas of life, discussing how individual behaviors (e.g., sheltering in place, keeping social distance, buying groceries online) and personal choices affected others during the pandemic (contributing or not to the spread of COVID-19).
COUPLEXP	Being in a relationship during the pandemic. Challenges and experiences (personal or friends) of relationships during the pandemic. Participants discussing experiences related to infidelity, communication, or wondering how people managed their relationships.
FEAROTHERHEALTH	Participant describing feeling anxious, worried, or fearful about a family member, friend’s health, or general concern during the COVID-19 pandemic.
FEARPERHEALTH	Feeling anxious, worried, or fearful about personal health during the COVID-19 pandemic.
LACKSOCRESP	Describing a lack of social responsibility/concern for others regarding the risk of coronavirus transmission/infecting others. Describing people not caring or considering others’ health during the COVID-19 pandemic.
NOCHANGE	People not making any changes in their lifestyles due to the COVID-19 pandemic.
NOFEAROTHERHEALTH	Participant sharing *not *feeling anxious, worried, or fearful about a family member, friend’s health, or others during the COVID-19 pandemic
NOFEARPERHEALTH	Participant sharing *not* feeling anxious, worried, or fearful about personal health during the COVID-19 pandemic
NOROMANTICREL	Describing *not* being involved (participant or friend) in a romantic or sexual relationship during the COVID-19 pandemic.
RISKYBEHAV	Describing engagement in risk taking behaviors during the pandemic. Risk taking behaviors included going to parties, drinking alcohol, and “hooking up.” Perceiving an increase in youth partying and opportunities to engage in risk taking behaviors. Describing reasons to engage in risk behaviors.
ROMANTICREL	*Involved* (participant or friend) in a romantic or sexual relationship during the pandemic, describing COVID-19 effects on romantic relationships.
SOCIALMEDIA	Role of social media on people’s lives during the pandemic. Use of social media for romantic or sexual relationships during the pandemic

From some, the COVID-19 pandemic and the shelter-in-place period were described as not interfering with romantic and/or sexual relationships. In such cases, the COVID-19 and shelter-in-place period was described as an opportunity to get to know their partners better, and to share more time together. In some cases, the COVID-19 pandemic period altered family dynamics surrounding youth relationships. For example, one participant shared that “I mean we’ve gotten closer. When we were quarantined, my grandma actually let him be quarantined with us. And yeah, he’s been staying over a lot more ever since the quarantine’s been happening like the whole pandemic.” To maintain intimacy during the shelter-in-place period, other youths described using digital media platforms when physical contact/togetherness was not possible. One participant who was no longer in a relationship at the time of the interview described the dynamic of former romantic relationship during the COVID-19 pandemic

Well me and my ex, we would like—We’d see each other once in a while but not every day, and like it would only be for like a certain amount of time and like we weren’t able to sit like really close together, we’d just have to like kind of sit near each other and like talk. Otherwise we’d just like text and stuff like that or call and call each other every day, text or like Snapchat and send pictures and whatnot, like anything. So like I’d text him and stuff like that always. Yeah, nothing really interesting, just texting and seeing each other once in a while.

In contrast, other youth described the COVID-19 pandemic as making it difficult to continue romantic involvement and maintain intimacy. Several described their relationships ending during that period due to communication problems and infidelity, which they attributed to COVID-19. For instance, one participant described how “My friend…her boyfriend I think cheated on her during that and they’re back together now, but I remember like last summer he cheated on her because like I think she was in quarantine actually and then he like went to some party and hooked up with some chick.” Another youth described how “I can say that a lot of my friends definitely have told me at one point or another that they were either done with the person or they liked someone else or dating someone.”

In addition, youth shared experiences about factors influencing romantic and/or sexual relationships during the COVID-19 pandemic such as concern for the wellbeing of others in their personal networks, and continued engagement in risk behaviors (going to parties and “hooking up”). Some youth expressed frustration about how others behaved irresponsibly by attending parties and engaging in risk taking behaviors that could have contributed to the spread of the virus, resulting in other people becoming sick. For example, one participant explained how

My friend…he lost his grandpa to COVID. And my friend, well my ex-best friend…they got together during COVID. Like one of their family members had COVID at the time anyways and I thought it was kind of like risky because they constantly were seeing each other and hugging each other and stuff like that.

Another explained that

Yeah, I just kinda sat there in awkward silence because like they both texted me at the same time and told me ‘Yeah, I’m with So-and-So now.’ I was like, ‘Oh, wait. Didn’t one of their family members have like COVID?’ They were like, ‘Yeah, but it’s okay. We don’t really see each other that often.’ I was like, ‘But you guys were around each other for the longest time and hugging and stuff like that.’ And they’re like, ‘Well we’re okay now.’ I was like, ‘Okay.’

Some participants continued sexual activity, but described a shifting social context directly related to the conditions of the shelter-in-place period. One participant described how:

In the beginning of COVID, we were all scared to even hang out with each other so I know that there wasn’t as much sexual, I feel like, or even, you know, romantic relationships with anyone. But towards the end, I noticed a lot more parties. I don’t go to parties myself, but yeah, I noticed a lot more parties.

Another described how “Because it was like growing up here, you already did everything, you already explored the creeks and stuff, nothing to do but now you’re teenagers and that stuff is more accessible to get drunk and drugs and stuff so everybody started doing it.” In addition, participants who continued having sex with their partners sometimes did so by staying in the same residence during the shelter-in-place period. One participant described how: “A bunch of people I knew were shacking up with a new person every week.” These behaviors could lead to outcomes that could influence attrition patterns. For example, one participant described a friend who continued to engage in sex with her romantic partner during the shelter-in-place period and who became pregnant and had a child as a result.

## Discussion

In this study of an ongoing SWD trial to evaluate an intervention to reduce sexual risk behavior among Assiniboine and Sioux youths, we found evidence of a secular trend occurring in relation to the COVID-19 pandemic period. Results have direct implications for informing future analyses at trial end, particularly the need for sensitivity analyses to determine whether clusters affected by the pandemic during the intervention period showed differential outcomes in comparison to clusters that received the intervention outside of the pandemic period.

First, we report quantitative and qualitative evidence suggesting the existence of a secular trend. The first cluster exposed to the intervention experienced school closures, social distancing, and sheltering-in-place orders between the finalization of the intervention and the 3-month post-intervention measurement period. Furthermore, individuals reporting having sex with less people due to COVID-19 reported an appreciably greater number of sex acts in comparison to those who did not report having sex with less people. Taken together, the results of this analysis suggest that more sexually active individuals reported perceiving having sex with less people and less frequent engagement in sex during the pandemic. This occurred between intervention completion and the first follow-up measurement. Qualitative evidence shows that youth continued to find ways to engage in sex despite the mitigation measures, which may have slightly decreased cumulative sexual activity during the trial. It is likely that the COVID-19 pandemic period was associated with a truncation in the distribution of otherwise normal sexual activity that would have occurred despite the existence of the intervention. While this could not be tested over time, cross-sectional evidence suggests it may be a possibility. Not accounting for this association might otherwise artificially inflate an estimate of the intervention’s effect at trial end. These findings will be informative to the specification of secular trends through sensitivity analyses that will be conducted at trial end and will also be informative toward the emerging discourse regarding scientific trials conducted in the context of CBPR with Indigenous communities.

Second, our results inform an understanding of internal validity regarding the use of cluster-randomized trials implemented in community settings during the pandemic. In SWD trials, clusters (as opposed to individuals) are the unit of randomization [32]. The randomization process increases internal study validity, perceived fairness, and transparency of allocation [33]. Altering the randomization sequence will undoubtedly raise questions about the cluster that moved its place in the sequence. The vulnerability of SWD trials to confounding effects underscores the importance of avoiding changes in intervention delivery to cohorts receiving the intervention and requires careful planning [34]. In one SWD trial where the non-random sequential assignment of clusters occurred due to logistical and ethical reasons [35], the study subsequently received criticism for its inability to control for secular trends in the outcome, and for undermining the estimation of an unbiased intervention effect [33]. In the context of our study, sensitivity analyses will be required to assess the full extent of the randomization sequence alterations described.

Finally, a large amount of attrition was observed in the first cluster randomized to the intervention. Indeed, it was logistically difficult to perform post-intervention follow-up observations with youth during the sheltering-in-place orders, changes in education delivery from in-school to remote based on parental requests, and school closures. However, attrition patterns did not vary from what might be otherwise a logical pathway to attrition (aging and/or graduating out of what would constitute membership in a cluster). Our current plans to address attrition include developing propensity scores for missing data at follow-up in a given observation period, matching cases, and repeating an imputation sequence for the models of interest [36]. Further analyses of attrition occurring for sites receiving the intervention during and after the pandemic will be required to understand how attrition patterns may vary relative to time.

### Limitations

This current study has limitations. First, the self-perceived effects of the COVID-19 pandemic on primary outcomes are cross-sectional and reported for a relatively small sample. It should be mentioned that the conditions of the pandemic limited normal logistics and operations of the research environment, and collecting these additional data was the appropriate option for *NE's* research team to mitigate risk and demonstrate flexibility and responsiveness given the conditions the COVID-19 pandemic created on the Fort Peck Reservation. Furthermore, our findings cannot be generalized to other SWD trials, but nonetheless, report exploratory data of use for similar trials where secular trends and internal validity will come into question. Finally, reporting on these findings may potentially increase the likelihood of observer bias associated with the trial, as researchers are now aware that a secular trend likely occurred for clusters exposed to the intervention during the pandemic. However, we still have not reported on trial efficacy to blind researchers from preliminary estimates of the intervention’s effectiveness.

## Conclusions

In this analysis of a SWD trial that evaluates SRH among Assiniboine and Sioux youth, we aimed to understand whether the COVID-19 pandemic may have produced a secular trend affecting the trial’s primary outcome. We reported quantitative associations between self-assessed impacts of COVID on sexual behaviors and self-reported sexual behaviors, and qualitative evidence of sex continuing despite limitations posed by COVID mitigation strategies. Together, results suggest evidence of a secular trend. Results will inform both sensitivity analyses and efforts to correctly specify the secular trend at trial end. Reporting these mid-trial findings encourages transparent reporting for unexpected events that occur during the implementation of a SWD trial during a global pandemic and will be informative for other cluster randomized controlled trials implemented in community settings.

## Supplementary Information

**Additional file 1.** Supplementary Materials.

## Data Availability

Data is available upon request.

## Abbreviations

CBPR: Community-based participatory research
COVID-19: Coronavirus disease 2019
NE: Nen ŨnkUmbi/EdaHiYedo (“We Are Here Now”)
SRH: Sexual and reproductive health
SWD: Stepped wedge design
