# Supplementary material for: Evidence of secular trends during the COVID-19 pandemic in a stepped wedge cluster randomized trial examining sexual and reproductive health outcomes among Indigenous youth
Source: Trials. 2023 Apr 1;24:248. doi: 10.1186/s13063-023-07223-1 (PMC10066013; doi:10.1186/s13063-023-07223-1)
Supplement: Supplementary file 1 — Additional file 1. Supplementary Materials. [file 13063_2023_7223_MOESM1_ESM.docx]

**Supplementary Materials**

S1. Variable importance measures (VIMs) for missing data at follow-up relative to baseline and mid-*NE* observation periods in first cluster randomized to the intervention for highest 10 VIMs in each period

S2. Probability of missing data at follow-up relative to age and grade of student among students in the fire cluster randomized to the intervention during baseline and mid-*NE* observations*

A. First cluster at baseline measurement, n=210 B. First cluster at mid-intervention measurement, n=145

**Probability values are derived from a logistic regression for missing data at follow-up, adjusted for a two-way interaction between age and grade*
